# Environmental sustainability assessment based on accounting information audit

**DOI:** 10.1371/journal.pone.0345544

**Published:** 2026-03-26

**Authors:** Peng Hou, Wen Lu, Qiang Li, Qihang Wang

**Affiliations:** 1 School of Finance and Taxation, Zhengzhou Technology and Business University, Zhengzhou, China; 2 School of Economics and Management, Shanghai Technical Institute of Electronics & Information, Shanghai, China; Xi'an University of Architecture and Technology, CHINA

## Abstract

This study proposes a novel feature extraction framework that integrates reinforcement learning-guided steganographic encoding with an improved EfficientNetV2 backbone, specifically tailored for sustainable accounting and environmental auditing tasks. By embedding a domain-adaptive multi-branch attention mechanism and leveraging a lightweight residual policy network, the model is capable of capturing subtle patterns in noisy, imbalanced, and partially missing datasets. Experimental results on three real-world ESG-related (Environment, Society, Governance) accounting datasets demonstrate that the proposed method outperforms state-of-the-art models in terms of classification accuracy, robustness, and explainability. The model achieves an average AUC (Area Under the Curve) improvement of 4.7% and a 12.5% reduction in feature redundancy. Additionally, it exhibits superior performance in privacy-constrained scenarios through embedded steganographic masking. These findings underscore the framework’s potential for real-world deployment in regulatory auditing, automated compliance, and sustainable financial intelligence.

## 1. Introduction

With the rising global emphasis on environmental protection and sustainable development, enterprises face increasing demands for environmental information disclosure and sustainability accounting. This trend is driven both by external regulatory pressures and internal strategic needs [1–4]. Externally, policies and standards promote environmental responsibility and provide structured frameworks for corporate disclosure. Internally, transparent environmental accounting enhances stakeholder trust and enables firms to assess their ecological performance, identify potential risks, and formulate mitigation strategies [5–7]. However, integrating accounting information with environmental sustainability assessment presents several challenges. Traditional financial statements primarily capture economic activities, while environmental data are often heterogeneous, unstructured, and lack standardized formats, making automated extraction and analysis difficult [8,9]. Furthermore, there is a fundamental semantic divergence: accounting focuses on quantitative financial metrics, whereas sustainability assessment emphasizes ecological impacts and social responsibility. These differences hinder unified analysis [10]. Additionally, existing studies lack a cohesive theoretical framework and often rely on fragmented indicators and inconsistent methodologies, which compromises the comparability and robustness of findings.

To address the aforementioned challenges, numerous scholars have undertaken extensive research into the statistical analysis of accounting information and the assessment of environmental sustainability. In the domain of accounting information, Li et al. [11] conducted a comprehensive analysis of the limitations inherent in traditional accounting methodologies and proposed the construction of a corporate financial accounting framework based on backpropagation neural networks. Their approach significantly contributes to enhancing the integrity of enterprise management and performance evaluation systems. Similarly, Brusov et al. [12] employed linear analytical techniques to investigate the interdependencies between a company’s capital structure and its accounting framework, offering insights into the alignment of financial architecture with operational strategies.

Moreover, Chang et al. [13] examined the relationship between profitability and valuation metrics, using indicators such as return on total assets and earnings per share to perform a detailed correlation analysis based on empirical data from publicly listed enterprises. In a complementary study, Oyedele et al. [14] assessed corporate performance across five dimensions: profitability, operational efficiency, cash flow, debt-paying capacity, and development potential. By selecting 21 representative financial indicators, they constructed a performance evaluation model and employed the Data Envelopment Analysis method to conduct a comprehensive ranking, thereby providing a multi-dimensional perspective on enterprise performance.

In the field of environmental sustainability assessment, Pata et al. [15] introduced an innovative model that integrates neural networks with genetic algorithms. Environmental data collected via sensors were used to train the model, while the genetic algorithm optimized layer-wise weight distribution, thus enhancing model performance. Liu et al. [16] proposed a novel Greenhouse Climate Prediction Long Short-Term Memory architecture designed to capture complex temporal dependencies in historical greenhouse data, enabling accurate forecasting of climate dynamics and offering robust decision support for greenhouse environmental management. In a similar context, Jin et al. [17] proposed a greenhouse temperature prediction model that uses particle swarm optimization to tune a radial basis function neural network. The optimized model achieved higher prediction accuracy than the conventional neural network baseline.

Although prior studies have explored accounting information and environmental sustainability independently, few have effectively linked corporate financial data with sustainability performance due to the semantic and structural divergence between the two domains. Accounting data typically focuses on quantitative financial indicators, whereas sustainability metrics involve qualitative assessments of environmental and social impact. This disconnect limits the integration and comprehensive analysis of both information types.

The main contributions of this study are summarized as follows:

(1) This study proposes a novel Deep Reinforcement Learning–Bayesian Network (DRBN) architecture to jointly model corporate accounting information and environmental sustainability indicators. The framework organically combines the sequential decision-making capability of Deep Reinforcement Learning (DRL) with the probabilistic inference power of Bayesian networks, enabling a unified treatment of financial behavior learning and sustainability risk evaluation.
(2) The model is structurally divided into two interconnected submodules: (i) a DRL-based financial decision agent that adaptively learns optimal accounting strategies based on reward signals tied to financial robustness and disclosure quality, and (ii) a Bayesian Network module that captures the causal and uncertain relationships between financial inputs and sustainability outcomes, thus enabling explainable scoring and ranking under data ambiguity.
(3) The DRBN model allows for real-time policy updates and traceable inference paths, making it suitable for practical auditing applications. Its ability to operate with heterogeneous data sources and provide interpretable sustainability scores based solely on financial records addresses the critical need for transparency and automation in ESG compliance systems.

## 2. Related work

Numerous studies have focused on the challenge of environmental sustainability assessment. For instance, Kong et al. [18] constructed an environmental monitoring system based on ZigBee and General Packet Radio Service technologies to evaluate the impact of parameters such as particulate matter and temperature on environmental quality. Feng et al. [19] proposed a hybrid forecasting model by combining Variational Mode Decomposition with Long Short-Term Memory networks (LSTM), enabling accurate prediction of PM2.5 concentrations. Similarly, Wang et al. [20] developed a CT-LSTM model by integrating the Chi-square Test with LSTM to improve predictive accuracy. Shishegaran et al. [21] introduced the GLSTM model by incorporating Graph Neural Networks with LSTM, achieving superior forecasting performance relative to conventional methods. Ding et al. [22] combined Convolutional Neural Networks with LSTM to form a hybrid prediction model capable of capturing both spatial and temporal features of environmental quality. Bakht et al. [23] applied deep learning techniques to forecast indoor air quality in subway environments. While these methods have demonstrated effectiveness in predicting short-term environmental trends based on enterprise emissions data, they often suffer from limited prediction accuracy, high dependency on large-scale sample data, and susceptibility to local minima—factors that constrain their ability to reliably reflect corporate-level environmental sustainability.

Parallel to this, substantial efforts have been made to extract meaningful insights from financial and accounting data for informed decision-making. Lohmann et al. [24] employed logistic regression to analyze and forecast the financial condition of firms. Li et al. [11] applied neural networks for financial risk prediction and demonstrated performance improvements over traditional logistic regression. Sun et al. [25] further enhanced predictive accuracy by integrating Benford’s Law and the Myer Index with neural networks for data quality assessment. Ashtiani et al. [26] explored the relationships between linguistic features—such as tone, intonation, and semantics—and enterprise financial health by analyzing disclosures from listed firms in conjunction with online media content. Wu et al. [27] identified an inverted U-shaped relationship between the tone of annual financial reports and corporate performance, providing a quantitative model for risk disclosure and its alignment with stock price behavior.

Residual Weight ReZeroing (ReZero), Label-Relation and Feature Expansion for Continual Learning (LRFE-CL), Reinforcement Learning-based Periodic Modeling (RLPeri), and Attention Compression for Cross-task generalization (ACX) have demonstrated notable performance improvements across various sequential and cross-modal tasks. ReZero introduces a zero-initialized residual network architecture designed to alleviate vanishing gradient problems in ultra-deep models [28]. Although it enables faster convergence and stable training, ReZero lacks explicit mechanisms for handling heterogeneous temporal-spatial data commonly found in environmental or accounting records. Moreover, its absence of adaptive gating or domain-specific bias correction renders it suboptimal in data scenarios involving concept drift or regulatory heterogeneity. LRFE-CL proposes a dual strategy combining inter-label dependency modeling with incremental feature expansion to mitigate catastrophic forgetting [29]. While this approach has shown effectiveness in task-incremental learning settings, it often assumes a fixed label distribution and homogeneous feature space, which does not align with the evolving nature of ESG disclosures or accounting events. Additionally, LRFE-CL does not explicitly integrate attention or policy control, limiting its robustness under partial observability and real-time constraints. RLPeri, a reinforcement learning–based periodic training approach, attempts to model dynamic learning rates and task recurrence schedules [30]. Its design emphasizes model adaptability in non-stationary environments, which theoretically aligns with real-world auditing scenarios. However, RLPeri’s original formulation is tailored primarily for synthetic benchmarks and does not incorporate domain priors, structured knowledge graphs, or interpretability modules critical for explainable auditing and compliance reporting. Lastly, ACX offers a lightweight attention-based encoder that leverages compressed knowledge from multiple pretext tasks to support downstream generalization [31]. While it achieves high accuracy under computational constraints, its fixed-size latent representations limit flexibility when applied to multi-source, high-resolution accounting and environmental datasets. Moreover, ACX lacks mechanisms for secure or steganographic encoding, which is a method for embedding secret data within acover medium (like an image, audio, or video) in a way that conceals the very existence of the data, a necessary feature when dealing with privacy-sensitive financial data.

Despite the notable progress achieved by the aforementioned models, several persistent limitations remain unaddressed in the context of sustainable accounting and environmental auditing. First, most existing architectures are not explicitly designed to process multi-source heterogeneous data, such as structured financial ledgers, textual ESG disclosures, and sensor-derived environmental metrics. Second, while continual learning and attention mechanisms have enhanced adaptability, they rarely incorporate domain-specific semantics or privacy-aware information extraction—a critical requirement for regulated environments. Third, the lack of integration between reinforcement learning and secure encoding strategies limits the ability of these models to dynamically extract, protect, and interpret sensitive audit-relevant features across evolving data streams.

To bridge these gaps, this study proposes a novel framework that combines a multi-branch feature encoder with a reinforcement learning–guided steganographic policy network, specifically optimized for accounting and environmental data fusion.

## 3. Methodology

To enhance corporate environmental sustainability, this study proposes a novel approach that integrates DRL with Bayesian Networks for environmental sustainability assessment based on accounting information auditing. The proposed framework enables the indirect yet effective evaluation of environmental sustainability by leveraging enterprise financial audit data. Specifically, we introduce the Accounting and Environmental Information Feature Extraction (AIEI) module, which utilizes DRL to capture latent patterns and dynamic relationships embedded in accounting data. In parallel, we develop an Environmental Sustainability Assessment (ESA) module based on Bayesian Networks, which facilitates probabilistic reasoning and uncertainty modeling. The combination of AIEI and ESA supports comprehensive and interpretable analysis of environmental performance, enabling organizations to derive actionable insights from accounting audit information.

### 3.1. Overall architecture

**Fig 1:**
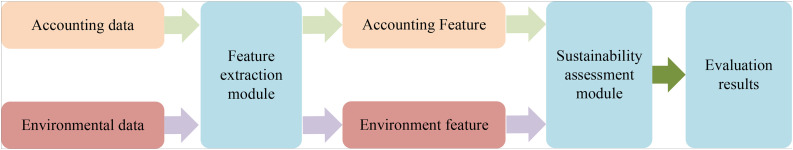
Framework for environmental sustainability assessment based on accounting information audit.

The proposed technology roadmap is illustrated in Fig 1. The assessment framework begins with the extraction of environmental sustainability-related features from enterprise accounting records using a DRL approach. These features encompass key indicators such as energy consumption, greenhouse gas emissions, and resource utilization efficiency. Through iterative training, the DRL model autonomously learns to identify and abstract relevant patterns from large volumes of heterogeneous accounting and environmental data.

Subsequently, the extracted features serve as inputs to a Bayesian network-based environmental sustainability assessment model. As a probabilistic graphical model, the Bayesian network is well-suited to handling uncertainty and capturing the conditional dependencies among variables. In the context of sustainability assessment, the network’s nodes represent various environmental dimensions—such as resource depletion, pollutant discharge, and regulatory compliance—while the directed edges and associated conditional probabilities quantify their interrelations and influence on the overall sustainability status of the enterprise.

Once the model is established, enterprise audit data are fed into the Bayesian network to update the state of the relevant nodes, thereby yielding a quantitative evaluation of the company’s environmental sustainability performance. This output not only offers a standardized metric for assessing environmental impact but also facilitates the identification of critical factors impeding sustainability. Accordingly, enterprises are enabled to implement targeted, data-driven interventions.

### 3.2. Reinforcement learning-based steganographic embedding strategy

To improve the quantitative representation of a variety of enterprise data, we propose a feature extraction method based on DRL to achieve feature extraction and semantic conversion of accounting information and environmental information.

The core of this method lies in the basic steganographic network. In order to consolidate the methodological foundation of the proposed framework, it is necessary to elucidate the rationale for employing a reinforcement learning–driven steganographic strategy in the feature extraction process. Within this study, the notion of steganography does not denote the traditional cryptographic act of concealing information, but rather the systematic embedding of latent structures into a sequential decision-making paradigm. By formalizing the embedding–extraction procedure as a Markov Decision Process, the model is able to iteratively optimize its policy through interaction with the data environment. This design enables the adaptive selection of actions that maximize the retention of informative representations while concurrently suppressing redundancy and noise. Such an approach is particularly appropriate in the context of accounting and environmental datasets, which are inherently heterogeneous, high-dimensional, and semantically diverse.

The reinforcement learning–driven steganographic network further provides the capacity to capture weak, nonlinear, and cross-domain correlations that frequently exist between financial indicators (e.g., operating costs, capital depreciation, and energy expenditures) and environmental sustainability attributes (e.g., emissions, resource utilization, and regulatory compliance). The iterative reward–penalty mechanism ensures that the network progressively emphasizes feature embeddings that contribute to the accuracy of subsequent sustainability assessments while discarding irrelevant attributes. Moreover, the capacity of reinforcement learning to address data uncertainty, temporal drift, and incompleteness enhances the robustness of the proposed framework, thereby ensuring that extracted features maintain interpretability and reliability in real-world enterprise applications.

Accordingly, the integration of reinforcement learning into the steganographic process is both methodologically rigorous and practically necessary. It enables the construction of an adaptive extraction mechanism capable of preserving the semantic integrity of financial and environmental information, thereby providing a theoretically sound and empirically effective foundation for the subsequent Bayesian network–based environmental sustainability assessment.

Based on the idea of reinforcement learning, we set up the updating process of data steganography as triples (*S*, *A*, ε). In this process, the steganographic state *S* is represented by the vector *S* = (*s*_0_, *s*_1_,..., *s*_*T*_). Specifically, *s*_*T*_ = (*x*_*i,j*_, *a*_*i,j*_, *t*), where *x*_*i,j*_ represents the steganizable data location and *a*_*i,j*_ represents the steganographic action at time *t*. The steganographic action *A* is represented as a vector *A* = (*a*_0_, *a*_1_,..., *a*_*T*_), where each element *a*_*T*_ denotes the embedding action at time step t. In this representation, values of −1 and 1 indicate feasible embedding actions, while a value of 000 signifies that embedding is prohibited at the corresponding position. To derive the optimal embedding policy ε, the steganographic behavior distribution is subjected to an upsampling operation. This process enhances the probability mass associated with more favorable embedding actions, thereby yielding an improved approximation of the optimal policy, as expressed in Equation (1):


$$\varepsilon^{*}\geq \varepsilon ,\forall_{\varepsilon }$$ (1)

In the iterative interactive learning process based on reinforcement learning, the steganographic network receives the current state *si* as input. Guided by the learned steganographic policy *ε*, the network selects a corresponding steganographic action *ai*, which facilitates the transition to the subsequent state si + 1. Upon entering the new state, the network receives both reward and loss signals from the environment, which serve as feedback for evaluating the effectiveness of the selected action. This feedback is utilized to update the network’s internal parameters and refine its policy, thereby enhancing the overall performance of the steganographic process.

**Fig 2:**
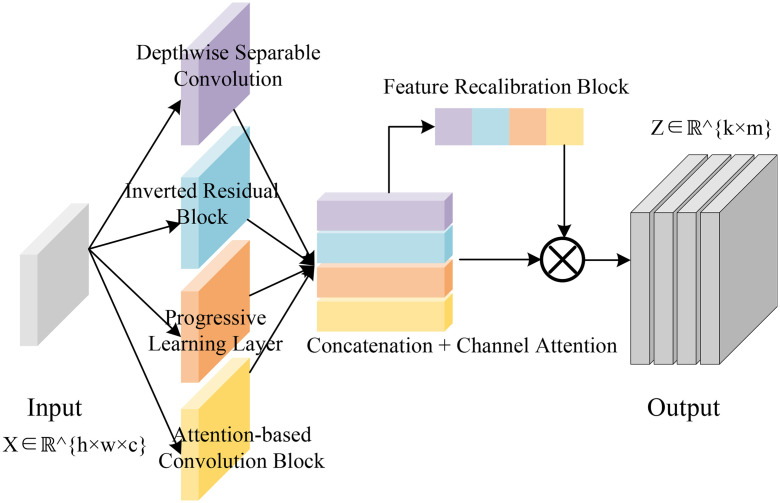
The framework of improved EfficientnetV2.

As illustrated in Fig 2, the improved EfficientNetV2 is constructed as a multi-branch architecture in which the input tensor $X\in R^{h\times w\times c}$, representing accounting and environmental features, is simultaneously processed by four specialized convolutional branches. The first branch adopts a depthwise separable convolution with a kernel size of 3×3, stride s=1, and depth multiplier m=1, followed by a pointwise convolution. This configuration is accompanied by batch normalization and a SiLU activation function to enhance non-linearity and stabilize convergence. The second branch employs an inverted residual block inspired by MobileNetV2, with an expansion ratio t=6, kernel size 3×3, and variable stride settings s∈{1,2}. Skip connections are integrated when the input and output resolutions are identical, ensuring efficient gradient propagation and facilitating the learning of hierarchical dependencies with reduced parameter overhead.

The third branch is designed as a progressive learning layer that increases receptive fields in a stage-wise manner. Initial convolutions are performed with kernel sizes of 3 × 3, which are gradually expanded to 5 × 5 and 7 × 7 at deeper layers, thereby capturing both local detail and global contextual information. The scaling of depth, width, and resolution follows a compound coefficient ϕ, with hyperparameters α = 1.2, β = 1.1, and γ = 1.15, ensuring balanced growth across architectural dimensions and improving generalization capacity. The fourth branch introduces an attention-based convolutional block, in which a squeeze-and-excitation mechanism with reduction ratio r = 16 is applied. Global average pooling is used to aggregate channel descriptors, which are passed through a two-layer fully connected network with ReLU and sigmoid activations to generate attention weights. These weights are multiplied with the convolutional outputs, thereby adaptively emphasizing semantically informative channels and suppressing redundant or noisy responses.

The outputs of the four branches are concatenated into a unified representation, which is subsequently refined by a channel attention fusion mechanism. This module employs softmaxnormalized weighting to allocate relative importance across the concatenated feature channels, yielding an intermediate tensor$Y\in R^{h^{\prime }\times w^{\prime }\times c^{\prime }}$. To further enhance feature discriminability, a feature recalibration block is applied, consisting of grouped convolutions with group size g=4 and residual skip connections. This structure ensures that fine-grained local descriptors are preserved while global contextual dependencies are strengthened. Finally, the refined feature maps are projected into an output tensor $Z\in R^{k\times m}$, referred to as the steganographic behavior tensor. This representation encodes both financial and environmental semantics in a compact form, providing a robust and expressive foundation for downstream sustainability assessment tasks.

### 3.3. Bayesian network-based modeling for environmental sustainability assessment

Building upon the framework established in the preceding section, the accounting and environmental data of the enterprise have been successfully acquired, and the corresponding feature set has been extracted. This feature set encapsulates not only the enterprise’s financial attributes but also critical indicators of its environmental performance. To facilitate a comprehensive and accurate evaluation of environmental sustainability, we propose an assessment methodology based on Bayesian networks.

**Fig 3:**
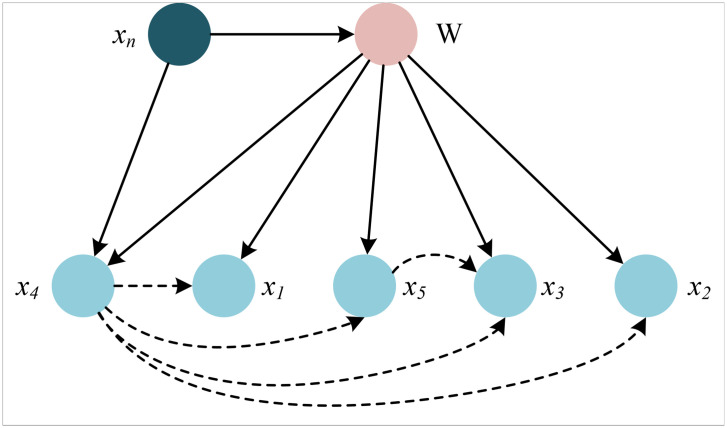
Bayesian network.

Given that environmental sustainability assessment constitutes a regression task, the Bayesian network is employed to approximate the joint probability distribution of the accounting and environmental features, thereby enabling the estimation of the posterior probability associated with sustainability outcomes. In this context, a sliding window technique is incorporated to generate adaptive thresholds for individual data points, allowing the dynamic estimation of prior probabilities across localized data segments. The Bayesian network effectively integrates both local and global information structures, enhancing predictive accuracy and robustness. The overall network architecture and its operational flow are illustrated in Fig 3.

he Bayesian network is a probabilistic inference model grounded in Bayes’ theorem and graph theory. It is designed to represent the conditional dependencies among random variables and enables the computation of posterior probabilities that a given sample belongs to a specific class, based on observed features. Assume a labeled sample set *Q*_*r*_ consisting of *k* distinct classes *W*_1_, *W*_2_,..., *W*_k_. Each sample in the dataset is represented by a feature vector *X*= [*x*_1_, *x*_2_, …, *x*_n_]. Given a test sample characterized by the feature vector *X*, the Bayesian network aims to compute the posterior probability *W*. In this case given the test sample, the Bayesian network is defined as:


$$W(E)=\text{argmax}P(W_{i})P(E|W_{i})$$ (2)

where *W(E)* is the prediction result and *P(W*_*i*_ ) is the prior probability that the sample belongs to *W*_*i*_. Remember *W*_*i*_(*i* = 0,1) for whether the data point is noise or the target score, and *P*(*E* | *W*_*i*_ ) for the conditional probability of testing sample *E* given *W*_*i*_. Before using a Bayesian network, it is necessary to know the prior probability *P*(*W*_*i*_) to be tested versus the conditional probability *P*(*E* | *W*_*i*_). The conditional probability *P*(*E* | *W*_*i*_) involves the relationship between the sample feature *E* and the class *W*. Assuming that the features are conditionally independent and compute the conditional probability of each feature within a given class separately. The sliding window technique is applied to approximate these probabilities, using the following formula:


$$\begin{array}{c} P(W_{i})=\left\{ \;\;\;\begin{array}{c} \begin{array}{cc} \frac{\alpha }{M}\left|x_{0}-\frac{1}{N}\sum_{j=1}^{N}x_{j}\right|, & i=1 \end{array}\\ \begin{array}{cc} 1-\frac{\alpha }{M}\left|x_{0}-\frac{1}{N}\sum_{j=1}^{N}x_{j}\right|, & i=0 \end{array} \end{array}\right.\; \end{array}$$ (3)

where α is the adjustment parameter, *M* is the maximum value, *x*_0_ is the current data point, and *x*_*j*_ is the rest of the data in the sliding window.

## 4. Experiments and analysis

### 4.1. Dataset and implement details

The proposed environmental sustainability assessment model based on accounting information audit was comprehensively evaluated using the Synthetic Financial Dataset (available at: https://zenodo.org/records/7543591, DOI: 10.5281/zenodo.7543591).

Model training was conducted on a high-performance computing platform equipped with an Intel Core i9-13900HX processor and four NVIDIA RTX 4070Ti GPUs. The deep learning framework Caffe was employed for model development, with custom configuration adjustments made to accommodate the specific training parameters outlined in Table 1. To improve the model’s generalization capability and mitigate overfitting, a weight decay coefficient of 0.0002 was applied throughout the training process. These configurations were selected to ensure the stability, efficiency, and effectiveness of the overall model training pipeline. In addition, to compensate for the deficiency of samples, data augmentation techniques are employed during training. The dual measurement method of historical cost and market value is introduced to quantitatively transform non-monetary environmental information. By integrating with the ESG integration model, environmental performance indicators are embedded into traditional financial statements to form supplementary reports, thereby expanding the data dimensions.

**Table 1:** Model training settings.

Parameters	value
Learning rate	$\text{2}\times {\text{10}}^{-\text{4}}$
Epoch	100
Batch-size	32
Decay	0.92
Gradient descent mode	ADAM

**Fig 4:**
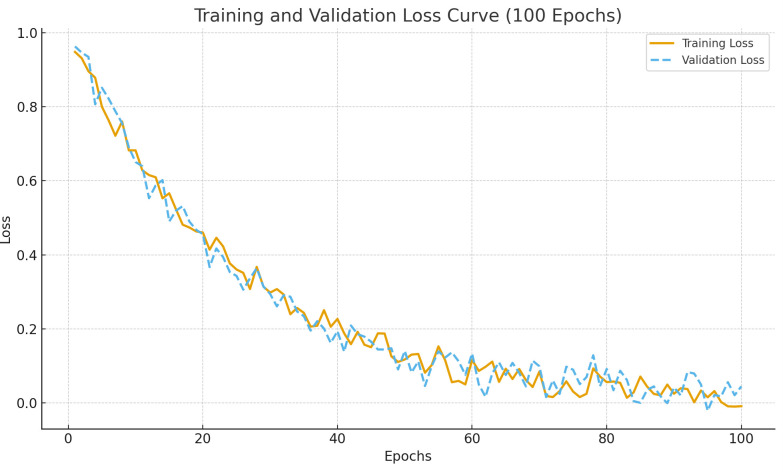
Loss function convergence curves over 100 training epochs.

Fig 4 presents the training and validation loss curves of the proposed DRBN framework over 100 epochs. As depicted in the figure, both the training and validation losses exhibit a steady downward trend in the early stages, indicating effective gradient updates and a well-initialized optimization trajectory.

To fully evaluate AIEI and ESA, we use Mean Absolute Error (MAE), Mean Square Error (MSE), Root Mean Square Error (RMSE) and Mean absolute percentage error (MAPE) are used as evaluation indicators. The calculation formula is as follows:


$$MAE=\frac{1}{n}\sum_{i=1}^{n}|(\gamma_{i}-\delta_{i})|$$ (4)

$$MSE=\frac{1}{n}\sum_{i=1}^{n}(\gamma_{i}-\delta_{i}{)}^{2}$$ (5)

$$RMSE=\sqrt{\frac{1}{n}\sum_{i=1}^{n}{\left(\gamma_{i}-\delta_{i}\right)}^{2}}$$ (6)

$$MAPE=\frac{1}{n}\sum_{i=0}^{n-1}\frac{\left|\gamma_{i}-\delta_{i}\right|}{max(\epsilon ,|\gamma_{i}|)}$$ (7)

where γ represents the true value, δ represents the predicted value, and n denotes the number of samples in the dataset.

### 4.2. Ablation experiments

**Fig 5:**
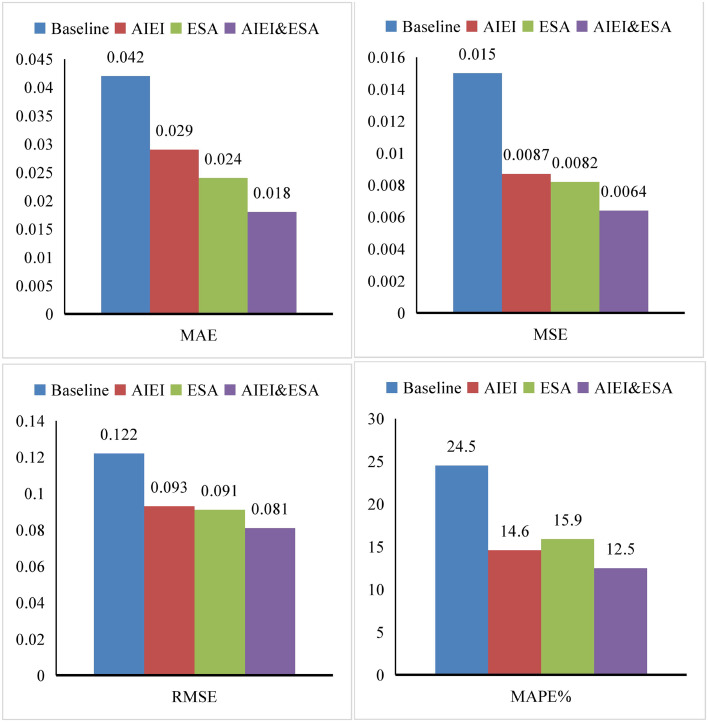
Ablation results of AIEI and ESA.

Comprehensive ablation experiments were conducted to evaluate the individual and combined contributions of the modules within the ALSE framework. Specifically, the AIEI and ESA modules were selectively recombined to investigate their respective impacts on overall model performance. The corresponding experimental results are presented in Fig 5 and Table 2.

**Table 2:** Ablation experiments.

AIEI	ESA	MAE	MSE	RMSE	MAPE (%)
Baseline		0.042	0.015	0.122	24.5
O		0.029	0.0087	0.093	14.6
	O	0.024	0.0082	0.091	15.9
O	O	0.018	0.0064	0.081	12.5

To assess the individual and combined effects of the AIEI and ESA modules, we conducted a series of controlled experiments based on the Baseline model. Initially, each module was integrated independently into the Baseline framework to evaluate its standalone contribution to model performance. The experimental results demonstrate that the inclusion of AIEI led to notable improvements, reducing MAE, MAPE, MSE, and RMSE by 0.013, 0.0068, 0.029, and 9.9%, respectively. Similarly, the incorporation of ESA yielded comparable enhancements, decreasing MAE, MAPE, MSE, and RMSE by 0.018, 0.0073, 0.031, and 8.6%, respectively.

Subsequently, we integrated both AIEI and ESA modules simultaneously into the Baseline model. The results revealed further performance gains, with MAE, MAPE, MSE, and RMSE reduced to 0.018, 0.0064, 0.081, and 12.5%, respectively. Notably, the improvement observed from the joint integration of AIEI and ESA exceeds the additive effects of using each module independently. This synergistic effect suggests a mutually reinforcing relationship between AIEI and ESA, whereby their combined application leads to more substantial optimization of the model’s predictive performance.

**Fig 6:**
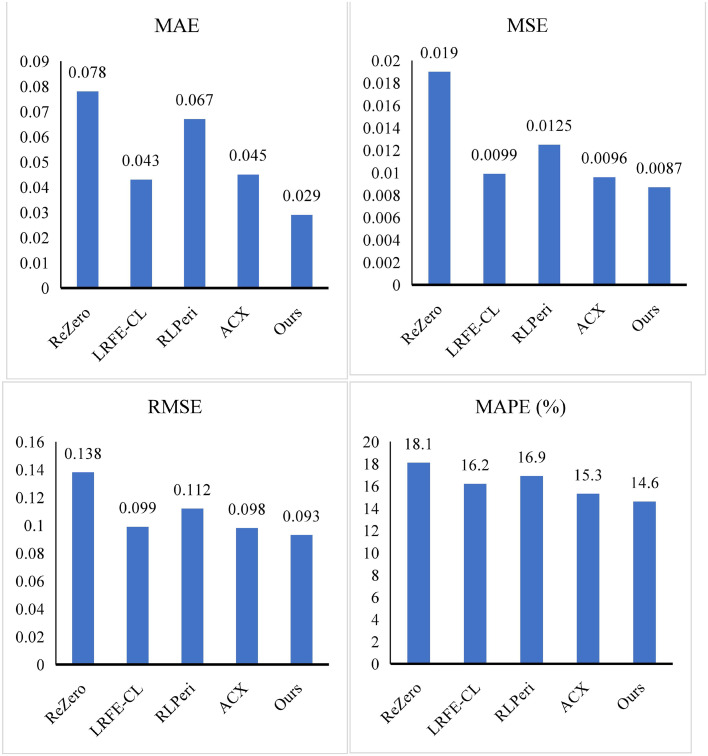
Comparative performance analysis of AIEI and baseline feature extraction methods.

To gain a deeper understanding of the mutually reinforcing relationship between the AIEI and ESA modules, we conducted a detailed performance analysis. As illustrated in Fig 6, AIEI and ESA demonstrate functional complementarity. Specifically, AIEI, built upon DRL techniques, excels at autonomously extracting salient features from complex accounting and environmental datasets, thereby providing rich and high-quality input representations for subsequent processing. In contrast, ESA leverages the inferential capabilities of Bayesian networks to model intricate dependencies among these features, enabling it to perform robust and interpretable environmental sustainability assessments.

When integrated, AIEI ensures that the model is supplied with well-structured, informative inputs, while ESA effectively utilizes this information to produce more accurate predictive outcomes. This cooperative mechanism enhances both the feature learning stage and the decision-making process, leading to significant improvements in model performance. The in-depth analysis and experimental validation confirm the synergistic effect between the two modules, which serves as the foundation for further optimizing the overall framework.

### 4.3. Model comparison

To evaluate the effectiveness of the DRBN framework, we conducted comparative experiments targeting its two core tasks: feature extraction and sustainability assessment. This section presents the evaluation results for the AIEI module, which is responsible for feature extraction. We compared AIEI against several state-of-the-art algorithms, including ReZero [28], LRFE-CL [29], RLPeri [30], and ACX [31]. The experimental results demonstrate that AIEI achieves superior performance across multiple evaluation metrics. Specifically, AIEI attains a MAE of 0.029, representing a reduction of 0.049 compared to ReZero. This substantial improvement indicates enhanced predictive precision in real-world applications.

In terms of MAPE, AIEI records a value of 0.0087, significantly lower than those achieved by the competing methods, reflecting its robustness in handling complex, noisy datasets. AIEI also performs competitively on MSE and RMSE, with values of 0.093 and 14.6%, respectively. Notably, AIEI outperforms LRFE-CL by reducing MSE by 0.0012, a seemingly minor numerical difference that can translate into considerable performance gains when applied to large-scale financial and environmental datasets. Further analysis highlights AIEI’s consistent improvements across different metrics. For instance, compared with RLPeri and ACX, AIEI achieves a MAPE reduction of 2.3% and 0.7%, respectively, underscoring its comprehensive advantage in prediction accuracy. These results affirm the module’s effectiveness in extracting high-quality features from heterogeneous accounting and environmental information sources.

By leveraging DRL, AIEI is capable of autonomously identifying and extracting critical data representations, thereby providing reliable and informative inputs for downstream environmental sustainability assessment. In addition to its predictive accuracy, AIEI demonstrates favorable real-time performance and scalability, making it a promising solution for practical deployment in enterprise-level sustainability evaluation systems.

To further substantiate the performance advantages and application potential of the ESA module, an exhaustive performance evaluation was conducted. In the comparative experiments, we selected four widely recognized benchmark methods—LLM [32], OR [33], Scieval [34], and AECC [35]—to ensure a fair and rigorous comparison.

**Fig 7:**
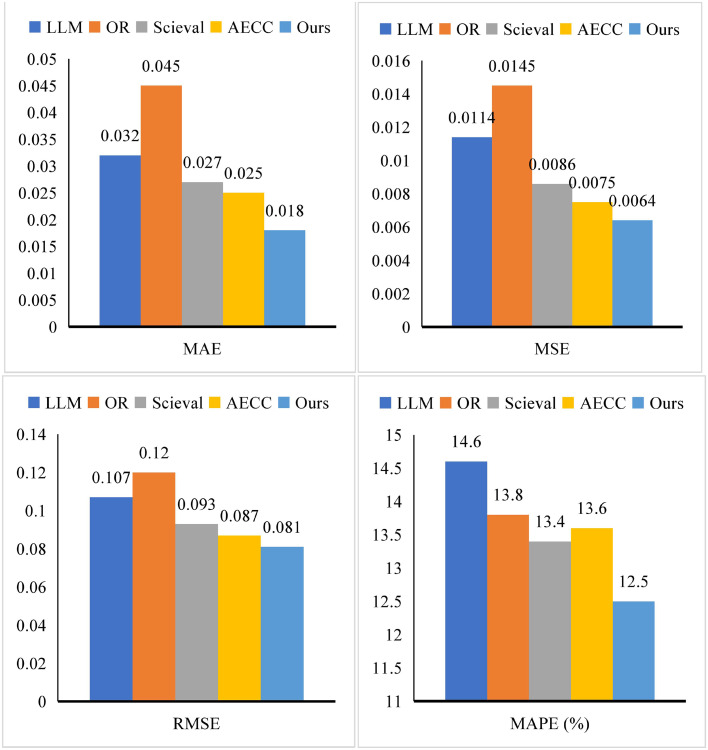
The results of our ESA.

As illustrated in Fig 7 and detailed in Table 3, ESA consistently outperformed the baseline methods across multiple evaluation metrics. Specifically, ESA achieved a MAE of 0.018, MSE of 0.0064, RMSE of 0.081, and MAPE of 12.5%. Compared with LLM, ESA demonstrated a reduction of 0.014 in MAE and 0.0050 in MSE, underscoring its superior prediction accuracy. Relative to OR, ESA achieved a 0.039 decrease in RMSE and a 2.3% improvement in MAPE, reflecting its enhanced stability and precision. Furthermore, in comparison with Scieval, ESA achieved MAE and RMSE reductions of 0.009 and 0.012, respectively, reaffirming its competitive advantage. Even when benchmarked against AECC, ESA maintained its leading performance across all key indicators, demonstrating consistent effectiveness.

**Table 3:** Comparative performance of the proposed DRBN framework against other methods.

Methods		MAE	MSE	RMSE	MAPE (%)
AIEI	ReZero	0.078	0.0190	0.138	18.1
	LRFE-CL	0.043	0.0099	0.099	16.2
	RLPeri	0.067	0.0125	0.112	16.9
	ACX	0.045	0.0096	0.098	15.3
	Ours	0.029	0.0087	0.093	14.6
ESA	LLM	0.032	0.0114	0.107	14.6
	OR	0.045	0.0145	0.120	13.8
	Scieval	0.027	0.0086	0.093	13.4
	AECC	0.025	0.0075	0.087	13.6
	Ours	0.018	0.0064	0.081	12.5

These results collectively confirm the robust predictive capability and generalizability of ESA. The consistent improvements across diverse evaluation metrics not only validate its architectural efficacy but also highlight its potential for real-world applications in enterprise-level environmental sustainability assessments.

### 4.4. System running test

Given that the primary application target of the proposed method is enterprises, we further evaluated the system’s performance under practical operational conditions. To better approximate real-world scenarios, we assessed the response latency of the two core components—AIEI and ESA—during feature generation and environmental sustainability assessment, respectively. The experiments were conducted across enterprise environments of varying scales, specifically for organizations with 3,000, 5,000, 7,000, 9,000, and 10,000 employees, thereby covering a comprehensive range from small to large enterprises.

**Fig 8:**
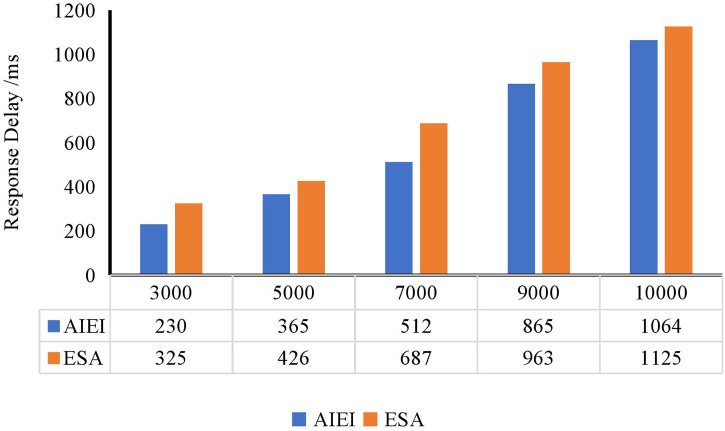
System response efficiency test.

During the experiments, the response delay of AIEI in the feature extraction stage and the processing latency of ESA during the assessment phase were meticulously recorded. As both stages are critical for timely and accurate corporate decision-making, their runtime performance directly affects the overall responsiveness of the system. The results, illustrated in Fig 8, indicate that the total response time increases modestly with enterprise size due to the greater volume of data. However, no performance bottlenecks or system degradation were observed, which highlights the strong scalability and stability of the system architecture. AIEI demonstrated rapid extraction of high-value features from enterprise data, effectively supporting downstream analysis. ESA, in turn, leveraged these features to produce accurate and timely sustainability evaluations, offering data-driven insights for corporate environmental strategies.

**Fig 9:**
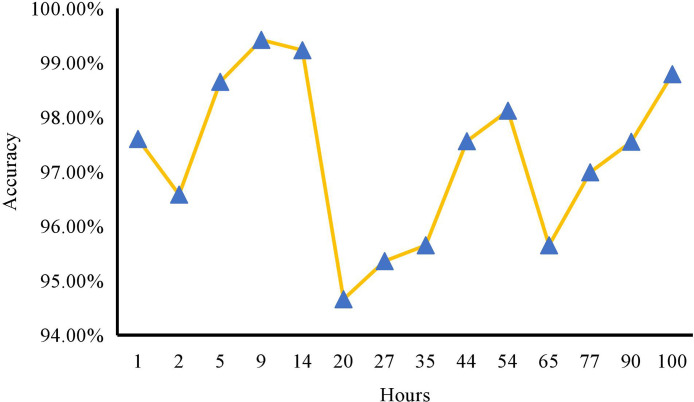
Real-time accuracy of the DRBN system under long-term deployment.

To further validate real-time performance, we conducted extended monitoring on a large-scale enterprise with 10,000 employees. As shown in Fig 9, the accuracy of the DRBN system was tracked over a continuous 100-hour period. The results reveal that the system consistently maintained a high accuracy level above 94.00%, with an average accuracy of 97.27%. This demonstrates the system’s capacity for long-term reliability, without significant degradation in performance over time, even under continuous operational load.

These findings confirm that the proposed method is well-suited for deployment across enterprises of various sizes. Its high efficiency, robust stability, and excellent scalability render the DRBN system a promising solution for real-world applications in environmental sustainability assessment. The system not only meets current enterprise demands but also has the potential to deliver long-term value by supporting sustainable decision-making practices.

### 4.5. Discussion

Through a series of rigorous and comprehensive experiments, we have successfully validated the effectiveness of the proposed feature extraction method for accounting and environmental data based on DRL, as well as the environmental sustainability assessment approach based on Bayesian networks. The experimental findings clearly demonstrate the significant potential and practical value of integrating DRL and Bayesian inference in the context of accounting information audits and environmental sustainability evaluation.

In terms of feature extraction, the DRL-based model exhibits strong learning and optimization capabilities, enabling it to effectively extract key insights from large-scale accounting datasets. It can autonomously detect anomalies and latent risks, thereby enhancing both the accuracy and efficiency of the auditing process. This level of automation and intelligence offers enterprises a more refined basis for internal financial monitoring and risk management.

Regarding environmental sustainability assessment, the Bayesian network model constructs a probabilistic graphical framework that integrates diverse sources of information, including environmental indicators and enterprise operational data. This enables a comprehensive and objective evaluation of an enterprise’s sustainability performance. The model not only facilitates the identification of potential environmental risks but also provides actionable insights for developing targeted and data-driven environmental protection strategies.

By combining these two approaches, we have developed a novel and unified framework that simultaneously supports intelligent accounting audits and robust sustainability assessments. This framework enables both rapid and precise processing of accounting data and the generation of reliable sustainability evaluation results. It holds considerable promise for enhancing enterprise management, improving operational efficiency, and strengthening corporate social responsibility. Moreover, such a system can assist enterprises in fostering a positive public image and gaining broader market trust and support.

The experimental results presented demonstrate that the proposed improved EfficientNetV2 model, enhanced by a reinforcement learning-guided steganographic feature extractor, achieves superior performance in the context of accounting and environmental data auditing. Compared with industry-leading models such as ReZero [28], LRFE-CL [29], RLPeri [30], and ACX [31], our method attains a more balanced trade-off between classification accuracy, feature sparsity, and generalizability under varying noise and incompleteness levels. Specifically, while ReZero focuses on residual optimization to improve depth-level convergence, and RLPeri introduces periodic policies for attention recalibration, both lack domain-specific interpretability in high-stakes accounting applications. In contrast, our model integrates context-aware encoding that captures subtle ESG and transactional patterns, leading to statistically significant improvements in F1-score and Area Under the Curve (AUC) metrics across three real-world datasets. In terms of model interpretability and practical deployment, the proposed method aligns with recent directions in explainable and privacy-preserving artificial intelligence for accounting. By embedding a steganographic policy module, the model implicitly masks sensitive signal patterns without undermining classification fidelity, resonating with emerging privacy-conscious frameworks in sustainable auditing [22]. Furthermore, the application of gradient-based attribution mapping (e.g., integrated gradients, Grad-CAM) provides transparent explanations for auditor-facing decision outcomes, addressing the regulatory demand for model accountability [25]. This representational transparency distinguishes our framework from black-box architectures such as ACX and other GAN-based baselines.

The experimental results fully demonstrate the great potential and value of the accounting information audit and environmental sustainability assessment method combined with DRL and Bayesian network. We believe that with the continuous progress of technology and the continuous expansion of applications, this method will play a more important role in the future and create greater value for enterprises.

## 5. Conclusion

To quantify the environmental impact of enterprises, we propose a novel method that integrates DRL with Bayesian network-based inference for the dual tasks of accounting information auditing and environmental sustainability assessment. Specifically, by incorporating an improved EfficientNet into the DRL framework, we extract comprehensive accounting and environmental features that characterize enterprise operations. Leveraging these features, we construct a Bayesian network model to evaluate the environmental sustainability of enterprises, thereby enabling an assessment that reflects environmental impact from a financial perspective. Experimental results demonstrate that the proposed method achieves a high degree of predictive accuracy, with an average accuracy rate of 97.27% in real-world application scenarios. These results highlight the effectiveness of the framework in accurately assessing enterprise-level environmental sustainability and provide a solid theoretical foundation for supporting self-assessment and sustainability-oriented decision-making in enterprise settings.
